# Sex differences in erythrocyte fatty acid composition of first-diagnosed, drug-naïve patients with major depressive disorders

**DOI:** 10.3389/fphar.2023.1314151

**Published:** 2023-12-18

**Authors:** Lu Wang, Ting Liu, Jimin Guo, Tingyu Zhao, Hui Tang, Fang Dong, Chuanyue Wang, Jindong Chen, Mimi Tang

**Affiliations:** ^1^ The National Clinical Research Center for Mental Disorders, Beijing Key Laboratory of Mental Disorders, Beijing Institute for Brain Disorders Center of Schizophrenia, Beijing Anding Hospital, Capital Medical University, Beijing, China; ^2^ Advanced Innovation Center for Human Brain Protection, Capital Medical University, Beijing, China; ^3^ National Clinical Research Center for Mental Disorders, Department of Psychiatry, China National Technology Institute on Mental Disorders, The Second Xiangya Hospital of Central South University, Changsha, Hunan, China; ^4^ Department of Pharmacy, Xiangya Hospital, Central South University, Changsha, Hunan, China; ^5^ National Clinical Research Center for Geriatric Disorders, Xiangya Hospital, Central South University, Changsha, Hunan, China; ^6^ College of Materials Sciences and Engineering, Beijing University of Chemical Technology, Beijing, China

**Keywords:** major depression disorder, erythrocyte membrane fatty acids, sex differences, n-6 PUFAs, n-3 PUFAs

## Abstract

**Background:** Since depression, sex hormones, and fatty acid status are interrelated, it is important to understand their relationships. In this study, we aimed to investigate sex differences in erythrocyte membrane fatty acid composition among first-diagnosed, drug-naïve patients with major depressive disorders.

**Methods:** The study included 139 individuals with first-diagnosed, drug-naïve depression (male/female = 48/91) and 55 healthy controls (male/female = 24/31). The levels of erythrocyte membrane fatty acids were analyzed to compare the difference between males and females in both patients with depression and healthy controls, as well as to study their correlation with depressive symptoms.

**Results:** In first-diagnosed, drug-naïve patients with major depressive disorders, sex disparities were observed in the levels of erythrocyte saturated fatty acids (SFAs) and n-6 PUFAs (such as C18:0, C20:4n6 and C22:4n6), where higher levels evident in females compared to in males. We found a noteworthy correlation between fatty acid levels and depressive symptoms, in which there is a significant association between female patients and depression but a weaker association between male patients and depression.

**Conclusion:** Our findings demonstrate higher levels of n-6 PUFAs and SFAs in female patients with depression. The relationship between fatty acid composition and depressive symptoms was more prominent in females than males. These findings highlight the significance of considering sex as a crucial and interconnected factor in future investigations and potential adjunctive treatment for mood disorders by targeting fatty acid metabolism.

## 1 Introduction

Major depressive disorder (MDD) is a severe mental disorder which is characterized by depressed mood and loss of appetite (McCarron et al., 2021). Females exhibit a higher risk of MDD compared to males. Data from the Global Burden of Disease (GBD) Study 2019 showed that the global prevalence rate was 3.0% in females, while it was 1.8% in males (GBD, 2019 Diseases and Injuries Collaborators, 2020). The proportion of total disability-adjusted life years (DALYs) attributed to MDD was 2.4% in females, while it was 1.35% in males (GBD, 2019 Diseases and Injuries Collaborators, 2020). Additionally, clinical characteristics and treatment responses of MDD patients also showed markable differences between females and males. Compared to males with depression, females with depression often experience earlier onset, lower quality of life, and increased comorbidity with anxiety disorder, somatization, and bulimia (Kornstein et al., 2000a; Marcus et al., 2005). Some studies indicated that females with depression exhibited a significantly favorable response to sertraline as an antidepressant compared to imipramine, while the opposite happened in males with depression (Kornstein et al., 2000b). It is notable that the peak of sex differences was reached at adolescence, and then declined and remained stable during adulthood (Salk et al., 2017). This implied the potential link between sex hormone fluctuations and depressive episodes and suggested the possible role of sex hormones in the onset, development, and treatment of depression (Shi et al., 2021).

Sex hormones are one of the crucial factors for essential fatty acid metabolism (Childs et al., 2008). Estrogen triggers the endogenous synthesis of polyunsaturated fatty acids (PUFAs). Studies showed that estrogen could upregulate the expression of delta-6 and delta-5 desaturase, as well as elongases (Extier et al., 2010; Kim et al., 2019), promoting the conversion of essential fatty acids to their longer-chain metabolites, especially PUFAs (Alessandri et al., 2008; Giltay et al., 2004a; Giltay et al., 2004b). On the contrary, testosterone has been associated with the downregulation of PUFA levels (Giltay et al., 2004a). Sex hormones and sex differences have been demonstrated in fatty acid status. Compared to males, females possess a higher proportion of PUFAs in plasma, including n-3 PUFAs (e.g., docose hexaenoie acid, DHA) and n-6 PUFAs (e.g., adonic acid, AA) (Burdge and Wootton, 2002; Lohner et al., 2013; Pawlosky et al., 2003). Moreover, compared to males, females exhibit a more efficient ability to replenish their n-3 PUFA status. These highlight the significant impact of sex hormones on fatty acid status (Extier et al., 2010).

The properties of lipid microdomains are considered biomarkers for individuals with depression (Liu et al., 2018), where the distribution of raft-associated proteins and the molecular architecture of lipid rafts are particularly vulnerable to modification by PUFAs (Simopoulos, 2011). In a previous study, we found a relationship between fatty acids and depressive symptoms and demonstrated that there are elevated levels of trans fatty acids (TFAs) and n-6 PUFAs in patients with depression (Wang et al., 2022). Notably, in patients with depression, the imbalance of fatty acids and depression symptoms could potentially be reversed by PUFAs or antidepressants (Kapoor et al., 2019).

Since depression, sex hormones, and PUFAs are interrelated, it is important to understand the sex differences in the fatty acid composition of patients with major depressive disorders. Previous studies showed that n-3 PUFA deficiency may contribute to the prevalence and prognosis of depression (Levant, 2013; Deacon et al., 2017; Tang et al., 2018). It is known that the levels of n-3 PUFAs are higher in females than in males (Lohner et al., 2013), however, the prevalence rate of depression is approximately 2-fold higher in females than in males (Beydoun et al., 2013; Colangelo et al., 2009). Additionally, some studies have shown that there is no association between the dietary intake of n-3 PUFAs and depression among males from a large cohort in Finland (Hakkarainen et al., 2004), while some epidemiological studies identified the associations between n-3 PUFA deficiencies and depressed patients particularly in females (Hamazaki et al., 2015; Smith et al., 2014; Yang et al., 2018). In this study, we further explore this topic and plan to investigate sex differences in erythrocyte fatty acid composition of first-diagnosed, drug-naïve patients with depression. To emphasize the relationship between fatty acid composition and depression, the inclusion criteria exclude individuals with prior antidepressant use and recurrent courses of depression. In addition, because the fatty acid status of erythrocyte membranes provides more reliable long-term assessments compared to those in serum or plasma (Assies et al., 2010; Carver et al., 2001), we will evaluate the fatty acid status of erythrocyte membranes to investigate the fatty acid metabolism *in vivo*. We aim to explore the relationships between sex differences in patients with depression and their fatty acid status, as well as their clinical symptoms.

## 2 Methods

### 2.1 Participants

The study was conducted at the Second Xiangya Hospital of Central South University from June 2017 to January 2020, which was approved by the Ethics Committee of the Second Xiangya Hospital of Central South University (MDD201610). All participants voluntarily participated in the study and signed informed consent.

The researchers randomly screened 139 first-diagnosed, drug-naïve depressed patients and 55 healthy controls (HCs) in the Mental Health Center of the Second Xiangya Hospital of Central South University. Data were drawn from the previous study exploring the relationship of fatty acids (FAs) composition with the presence and clinical characteristics of first-diagnosed, drug-naïve patients with depression; results of these studies have been previously published (Wang et al., 2022). Each patient was evaluated by two experienced psychiatrists for a diagnosis of depression according to the DSM-5 (Diagnostic and Statistical Manual of Mental Disorder, fifth version). All included patients had HAMD scores greater than 21 points. The inclusion and exclusion criteria of patients were described in the previous study (Wang et al., 2020). Healthy controls (HCs) included in the study had no prior or current diagnosis of any psychiatric disorder. HCs with the following conditions were excluded: 1) severe medical and chronic diseases; 2) daily use of benzodiazepines; 3) history of psychoactive substance abuse (except alcohol and tobacco); 4) women who were pregnant or lactating or had pregnancy plans during the trial period; 5) Any conditions or drugs that may affect biomarkers. Patients and HCs with similar gender, age, and education year.

### 2.2 Clinical assessment

General and clinical data of each participant including gender, age, body mass index (BMI), marital status, fertility condition, living and growing environment, education year, family history, and other data were carefully recorded. All patients were assessed by 24-item HAMD (24-item Hamilton Depression Scale) and Hamilton Anxiety Scale (HAMA) interviews, and patients and HCs were required to complete the Self-Rating Anxiety Scale (SAS), and Beck Depression Inventory (BDI) assessments. Both psychiatrists involved in the assessment had more than 5 years of clinical experience and had been trained in standardized scale assessment before the assessment. Furthermore, to maintain high interrater reliability, the scale data was supervised by a chief psychiatrist and two psychiatrists met at least once a month for training and reliability re-testing during recruitment.

### 2.3 Sample collection and analysis

All participants collected venous blood in the morning without eating. After the blood samples were centrifuged at 3,000 rpm for 10 min, the plasma-removed erythrocytes were collected and stored in a −80°C refrigerator. The lipid extraction method and analysis procession as described in the previous study (Li et al., 2022). The concentration unit of fatty acids (FAs) was mmol/L, and some indicators were expressed in relative content or ratio. n-3 PUFAs were defined as C20:5n3 + C22:5n3 + C22:6n3; n-6 PUFAs were defined as C18:2n6c + C20:3n6 + C20:4n6 + C22:4n6; Total PUFAs was the sum of n-3 PUFAs and n-6 PUFAs. The calculation formula of the n-3 index was the ratio of [eicosapentaenoic acid (EPA, 20:5n3) + DHA (22:6n3)]/FAs; The n-6/n-3 ratio was used to assess the balance of n-6 and n-3 PUFAs. The elongation process of n-6 and n-3 was evaluated using the ratios of C22:4n6/C20:4n6 and C22:5n3/C20:5n3 respectively as previously described (Li et al., 2022). The ratio of FA product to precursor was defined as the desaturase index, which can be used to estimate desaturase activity. In this study, the ratio of C18:1/C18:0 was used to assess stearoyl-CoA desaturase-1 (SCD-1) activity, C20:3n6/C18:2n6 to assess D6 desaturase activity, and C20:4n6/C20:3n6 to assess D5 desaturase activity.

### 2.4 Statistical analysis

Data analyses were performed by IBM SPSS Statistics version 26.0 software and R 4.1.3. The choice of statistical methods is based on the type of data and the purpose of the analysis. All continuous variables were examined for normality by the Kolmogorov-Smirnov test. Data following normal distribution were analyzed using an independent sample *t*-test and presented as mean (SD); Non-normal data were examined using the Mann-Whitney U Test and expressed as the interquartile range (Q1–Q3). The chi-square test or Fisher’s exact probability method was used for the statistical analysis of enumeration data. The normal distribution of FAs data was examined by two-way analysis of variance (ANOVA) to examine the main effects and interactions of diagnosis and sex differences; non-normal FAs data were examined by Scheirer-Ray-Hare test with Bonferroni correction used to adjust for multiple comparisons. Spearman correlation analysis was used to test the correlation between depression and anxiety symptoms and erythrocyte FA composition, and multiple linear regression was used to correct the influence of confounding factors. The potential confounders that have an impact on the relationship between fatty acid levels and depression based on previous research as well as clinical importance to enter into the multivariate model (Féart et al., 2008; Jadoon et al., 2012). *p* < 0.05 was considered statistically significant.

## 3 Results

### 3.1 Clinical and demographic characteristics

The clinical and demographic data of HCs and depressed patients are shown in Table 1. A total of 139 patients and 55 healthy controls were included in the study. Depressed patients included 48 males and 91 females; HCs included 24 males and 31 females. Patients and HCs had significant differences in marital status (*p* < 0.001), only child (*p* = 0.012), and family history (*p* = 0.023). The BDI (*p* < 0.001), and SAS (*p* < 0.001) scores of patients were significantly higher than those of HCs. There were significant differences between males and females in the current smoking (*p* < 0.001) and current drinking (*p* = 0.048) in patients, while in HCs, males had fewer years of education (*p* = 0.042) and most had a history of smoking (*p* = 0.035).

**TABLE 1:** Clinical and demographic characteristics of patients with MDD and HCs.

	MDD *n* = 139			HCs *n* = 55			
	Males *n* = 48	Females *n* = 91	*p* value	Males *n* = 24	Females *n* = 31	*p* value	Group (*p*-value)
Age (years)	27.17 (7.90)	27.80 (9.08)	0.682	29.88 (9.06)	28.52 (8.77)	0.577	0.273
Education (years)	14.00 (2.53)	13.71 (3.46)	0.627	13.21 (2.26)	14.81 (3.18)	**0.042**	0.557
Fertility status	13/35	35/56	0.180	10/14	13/18	0.984	0.342
Marital status	13/35	34/57	0.223	14/9	17/12	0.921	**<0.001**
Only child	20/27	33/56	0.534	4/20	7/24	0.838	**0.012**
Family history	13/35	19/70	0.449	1/23	4/27	0.373	**0.023**
Current smoking	15/33	6/85	**<0.001**	6/18	1/30	**0.035**	0.671
Current drinking	5/43	2/89	**0.048**	0/24	0/31	1.000	0.194
BMI	21.84 (3.10)	21.18 (2.72)	0.220	22.76 (2.58)	21.42 (2.68)	0.068	0.198
HAMD	29.63 (6.67)	29.80 (6.58)	0.888	-	-	-	-
HAMA	21.63 (7.02)	23.17 (6.46)	0.205	-	-	-	-
BDI	26.93 (10.4)	29.30 (9.84)	0.198	7.708 (8.87)	6.194 (6.88)	0.478	**<0.001**
SAS	43.20 (8.39)	44.99 (9.28)	0.281	32.79 (6.26)	31.26 (6.01)	0.361	**<0.001**

BMI, body mass index; BDI, beck depression inventory; HAMA, hamilton anxiety scale; HAMD, 24-item Hamilton Depression Rating Scale; HCs, healthy controls; MDD, major depressive disorder; SAS, Self-rating Anxiety Scale.

The data are expressed as mean ± SD.

Bold represents a statistically significant difference.

### 3.2 Fatty acids of erythrocyte membrane in the male and female subjects in different groups

As shown in Table 2, the two-way ANOVA results showed that depression significantly affected C18:1n9t (F = 3.859, df = 1, *p* = 0.049), C18:1n9c (F = 10.57, df = 1, *p* = 0.001), C20:3n6 (F = 5.654, df = 1, *p* = 0.019). Among the biomarkers, the level of C18:1n9c (*p* = 0.004) in male HCs was significantly higher than that in male patients, while the level of C20:3n6 (*p* = 0.012) in female patients was significantly higher than that in female HCs. In addition, C18:0 (F = 4.201, df = 1, *p* = 0.040), C20:4n6 AA (F = 4.805, df = 1, *p* = 0.028), C22:4n6 (F = 4.161, df = 1, *p* = 0.041) had significant gender differences in two groups. Among them, C20:4n6 AA (*p* = 0.008) and C22:4n6 (*p* = 0.006) were significantly higher in female patients than in males. Moreover, there was an interaction between diagnosis and gender on the effect of the C22:5n3/C20:5n3 ratio.

**TABLE 2:** Fatty acid composition of male and female subjects in different groups.

	HCs *n* = 55		MDD *n* = 139		Diagnose F/H (*p* value)	Sex F/H (*p* value)	Diagnose + sex F/H (*p* value)
	Male n = 24	Female n = 31	Male n = 48	Female n = 91			
C16:0	7.45 (4.93–13.9)	7.25 (4.95–12.0)	7.85 (5.71–10.7)	10.6 (5.93–13.9)	2.575 (0.109)	1.409 (0.235)	1.292 (0.256)
C18:0	3.29 (2.66–9.07)	5.21 (2.58–8.99)	4.81 (3.21–6.36)	5.74 (4.04–8.18)	1.263 (0.261)	**4.201 (0.040)**	0.012 (0.912)
C18:1n9t	4.04 (2.55–7.91)	5.10 (2.91–7.43)	5.70 (3.92–7.90)	6.28 (4.37–7.90)	**3.859 (0.049)**	0.255 (0.614)	0.033 (0.857)
C18:1n9c	0.58 (0.16–0.60)^#^	0.49 (0.14–0.61)	0.29 (0.13–0.36)	0.28 (0.10–0.42)	**10.57 (0.001)**	0.057 (0.811)	0.709 (0.400)
C18:2n6c	3.70 (2.14–5.49)	3.20 (2.27–5.02)	3.47 (2.69–5.49)	4.17 (3.27–6.57)	3.024 (0.082)	1.536 (0.215)	1.787 (0.181)
C20:3n6	0.25 (0.13)	0.24 (0.11)^#^	0.28 (0.11)	0.31 (0.13)	**5.654 (0.019)**	0.152 (0.697)	0.754 (0.387)
C20:4n6 AA	2.75 (1.44–6.03)	3.45 (1.69–5.61)	3.45 (1.67–4.67)*	4.53 (3.07–7.12)	2.053 (0.152)	**4.805 (0.028)**	1.987 (0.159)
C20:5n3 EPA	0.03 (0.02–0.08)	0.05 (0.02–0.08)	0.05 (0.03–0.07)	0.06 (0.03–0.08)	1.161 (0.281)	1.177 (0.278)	0.053 (0.817)
C22:4n6	0.56 (0.27–1.03)	0.53 (0.30–0.97)	0.56 (0.26–0.84)*	0.84 (0.52–1.14)	1.507 (0.220)	**4.161 (0.041)**	2.865 (0.091)
C22:5n3	0.34 (0.17–0.80)	0.38 (0.18–0.66)	0.49 (0.20–0.70)	0.59 (0.35–0.84)	3.156 (0.076)	2.114 (0.146)	1.408 (0.235)
C22:6n3 DHA	0.79 (0.26–1.17)	0.67 (0.23–1.13)	0.65 (0.24–0.99)	0.96 (0.57–1.36)	0.867 (0.352)	3.754 (0.053)	2.571 (0.109)
Total FAs	23.0 (15.8–40.3)	25.3 (15.1–42.7)	29.0 (18.9–36.9)	37.2 (23.2–45.3)	2.774 (0.100)	2.203 (0.138)	0.975 (0.323)
Total SFAs	10.8 (7.16–22.9)	11.5 (7.33–21.6)	12.9 (8.99–16.4)	16.9 (9.98–22.0)	1.995 (0.158)	2.385 (0.122)	0.513 (0.474)
Total MUFAs	4.42 (2.91–8.54)	5.60 (3.08–7.75)	5.89 (4.24–8.32)	6.64 (4.70–8.31)	2.889 (0.089)	0.195 (0.659)	0.100 (0.752)
Total PUFAs	8.28 (4.75–15.1)	8.83 (4.76–13.9)	9.94 (6.71–12.4)	11.5 (8.15–17.3)	2.711 (0.100)	3.726 (0.054)	2.311 (0.128)
n-6	7.35 (4.26–13.3)	7.60 (4.34–12.1)	8.75 (6.23–10.5)	10.1 (7.08–14.2)	2.838 (0.092)	3.478 (0.062)	2.264 (0.132)
n-3	1.24 (0.45–2.10)	1.05 (0.45–2.05)	1.22 (0.46–1.80)	1.56 (0.94–2.31)	1.896 (0.169)	3.198 (0.074)	2.141 (0.143)
n-6/n-3	7.72 (5.59–9.76)	8.19 (5.59–10.2)	8.08 (5.63–9.97)	7.41 (5.53–9.90)	0.002 (0.967)	0.173 (0.677)	0.660 (0.417)
UI	1.31 (0.23)	1.28 (0.20)	1.26 (0.26)	1.38 (0.24)	0.313 (0.577)	1.147 (0.286)	3.312 (0.071)
(EPA + DHA)/total FAs	0.02 (0.02–0.04)	0.02 (0.02–0.04)	0.02 (0.02–0.03)	0.03 (0.02–0.03)	<0.001 (0.991)	0.831 (0.362)	1.807 (0.179)
C18:0/C16:0	0.62 (0.48–0.68)	0.63 (0.50–0.75)	0.56 (0.47–0.66)	0.57 (0.52–0.70)	1.198 (0.274)	1.915 (0.166)	0.004 (0.951)
C18:1n9/C18:0	1.03 (0.93–1.27)	1.05 (0.92–1.20)	1.18 (0.97–1.41)	1.11 (0.92–1.31)	3.831 (0.050)	3.061 (0.080)	0.047 (0.828)
C20:3n6/C18:2n6	0.06 (0.03–0.09)	0.06 (0.03–0.11)	0.07 (0.03–0.13)	0.06 (0.03–0.09)	0.084 (0.772)	0.313 (0.576)	0.254 (0.614)
C20:4n6/C20:3n6	16.38 (8.63)	17.43 (8.98)	15.19 (9.38)	17.84 (8.89)	0.064 (0.801)	1.409 (0.237)	0.263 (0.608)
C22:4n6/C20:4n6	0.18 (0.15–0.20)	0.18 (0.15–0.21)	0.18 (0.13–0.19)	0.17 (0.14–0.19)	2.554 (0.110)	<0.001 (0.990)	0.175 (0.676)
C22:5n3/C20:5n3	10.9 (6.68–15.3)	7.62 (5.62–10.8)^#^	9.24 (6.45–12.8)	11.4 (7.41–16.0)	1.759 (0.185)	0.187 (0.665)	**5.384 (0.020)**

AA, arachidonic acid; DHA, docosahexaenoic acids; EPA, eicosapentaenoic acid; FAs, fatty acids; HCs, healthy controls; MDD, major depressive disorder; MUFAs, monounsaturated fatty acids; PUFAs, polyunsaturated fatty acids; SFAs, saturated fatty acids; UI, unsaturation index.

The asterisk symbol indicates the comparison between males and females in patients or healthy controls: *p < 0.05.

The plus sign indicates the comparison between patients and healthy controls in males or females: ^#^ *p* < 0.05.

The unit of concentration of each fatty acid was mmol/L.

a: The data are expressed as mean ± SD; b: The data is expressed as the interquartile range (Q1-Q3).

Bold represents a statistically significant difference.

### 3.3 Association between fatty acids of erythrocyte membrane and scale scores

**FIGURE 1:**
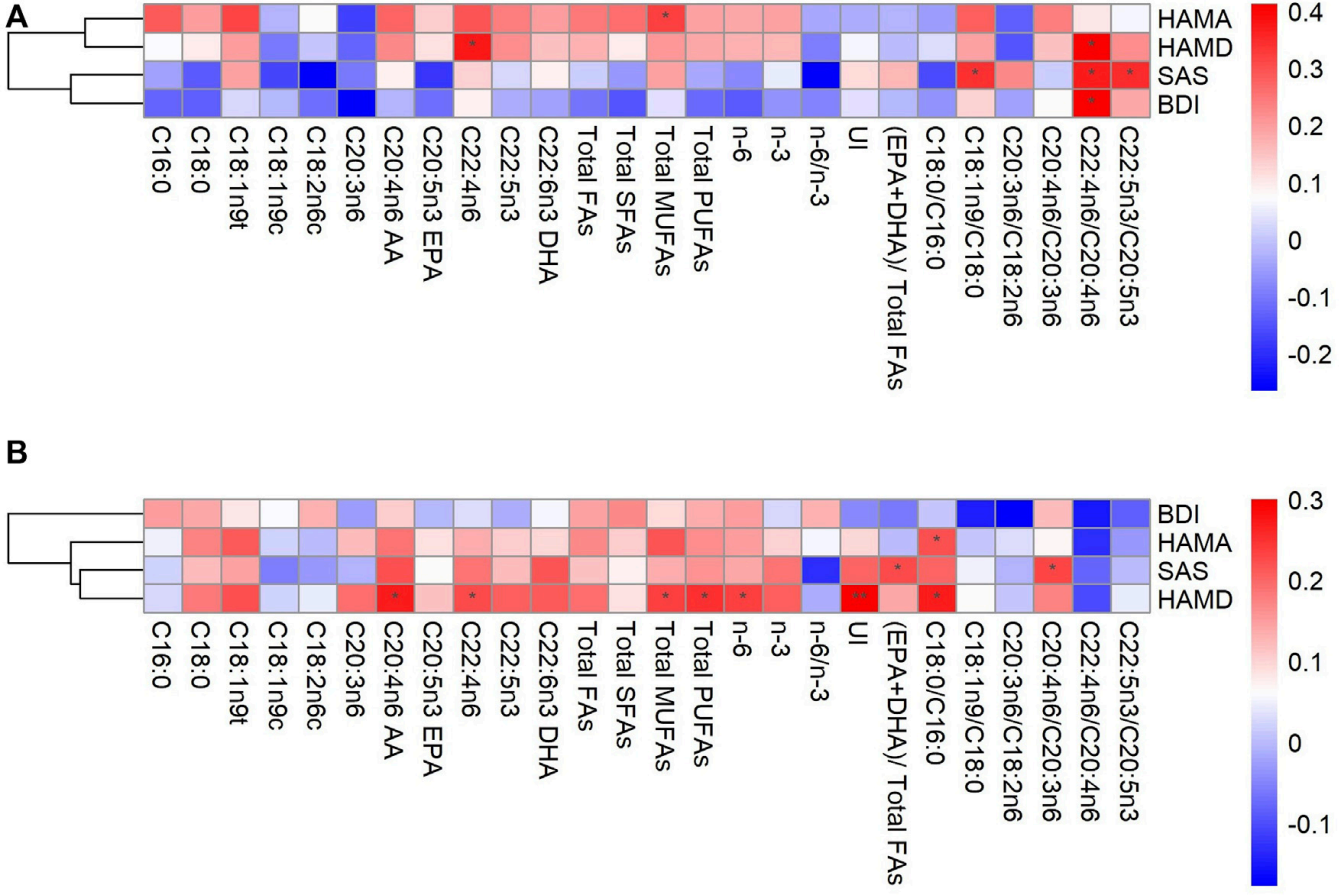
Correlations between fatty acids and depression in the male **(A)** and female **(B)** AA, arachidonic acid; BDI, Beck Depression Inventory; DHA, docosahexaenoic acid; EPA, eicosapentaenoic acid; FAs, fatty acids; HAMA, Hamilton Anxiety Scale; HAMD, 24-item Hamilton Depression Rating Scale; MUFAs, monounsaturated fatty acids; PUFAs, polyunsaturated fatty acids; SFAs, saturated fatty acids; SAS, Self-Rating Anxiety Scale; UI, unsaturation index, *p < 0.05, **p < 0.01.

As shown in Figure 1A, C22:4n6, Total MUFAs, C18:1n9/C18:0, and C22:5n3/C20:5n3 levels were associated with HAMD, HAMA, and SAS scores in male patients, respectively (*p* < 0.05). While the level of C22:4n6/C20:4n6 was significantly positively correlated with HAMD, BDI, and SAS scores (*p* < 0.05). Overall, most FAs and BDI, SAS scores showed a negative correlation trend. Unlike males, All the scores on other scales showed a positive trend with FAs in female patients (Figure 1B). Specifically, HAMD score was positively correlated with C20:4n6 AA, C22:4n6, Total MUFAs, Total PUFAs, n-6 PUFAs, and C18:0/C16:0 (*p* < 0.05), and UI showed a strong positive correlation (*p* < 0.01). C18:0/C16:0 and HAMA also showed a positive correlation (*p* < 0.05). In addition, (EPA + DHA)/total FAs, C20:4n6/C20:3n6, and SAS showed a significant positive correlation (*p* < 0.05).

### 3.4 Association between fatty acids and depressive symptoms in the multivariable model

**FIGURE 2:**
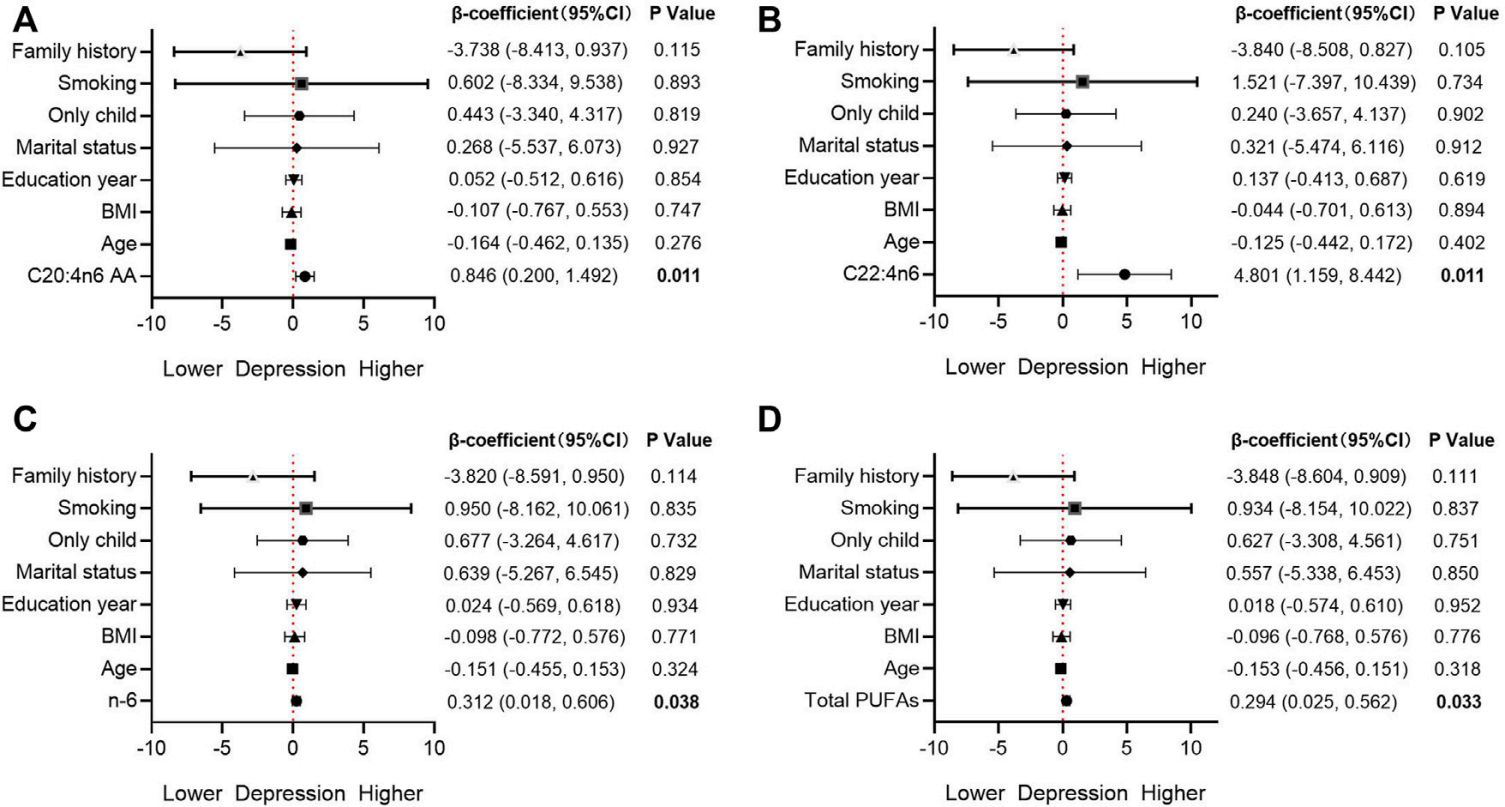
Association between fatty acids and depression in the multivariable model AA, arachidonic acid; BMI, body mass index; CI, confidence interval; PUFAs, polyunsaturated fatty acids.

The FA indices with significant differences in Spearman correlation were included in the multiple linear regression model (Figure 2). After adjusting age, BMI, education year, marital status, only child, smoking and family history, C20:4n6 AA (*β* = 0.846, *p* = 0.011, adjusted R^2^ = 0.102), C22:4n6 (*β* = 4.801, *p* = 0.011, adjusted R^2^ = 0.103), Total PUFAs (*β* = 0.294, *p* = 0.033, adjusted R^2^ = 0.069), and n-6 (*β* = 0.312, *p* = 0.038, adjusted R^2^ = 0.064) were still significantly correlated with the depressive symptoms.

## 4 Discussion

Our results demonstrate that the relationship between fatty acid composition and depressive symptoms was more pronounced in females than males. Compared to males with depression, there are higher levels of n-6 PUFAs [C20:4n6 (AA) and C22:4n6] and saturated fatty acid [C18:0 (SFAs)] in the depressed female population. Consistent with previous studies, higher proportions of AA or other types of n-6 PUFAs have been linked to depression (Hoge et al., 2019; Lohner et al., 2013; Vaz et al., 2014). In females, higher levels of estrogen trigger the endogenous synthesis of PUFAs. n-6 PUFAs are considered to have proinflammatory properties (Saini and Keum, 2018). By altering serotonin metabolism and reducing synaptic plasticity, pro-inflammatory n-6 PUFAs might increase the risk of developing depression, a chronic low-level inflammatory disease for a long time (Beurel et al., 2020; Myint and Kim, 2003; Zheng et al., 2021). Additionally, reactive oxygen species could be induced by overconsumption of SFAs, which leads to the progression of depression (Nakamura et al., 2019; Bhatt et al., 2020; Marx et al., 2021). Our study revealed that levels of n-6 PUFAs and SFAs were negatively associated with depressive symptoms in female patients with MDD. The higher levels of n-6 PUFAs and SFAs may be one of the risk factors for the development of depression in females. This finding may provide valuable biomarkers for depression diagnosis and identifying new targets for treatment development.

Contrary to previous studies which suggested there was a relationship between n-3 PUFA deficiency and depression (Messamore et al., 2017; Morgese et al., 2020), in our study, there are no significant changes in n-3 PUFAs both in health control and patients with depression. Furthermore, our study shows that levels of n-3 PUFAs in depressed females are even slightly higher than in healthy females and depressed males, which does not match with previous epidemiological studies that n-3 PUFAs are associated negatively with depressive symptoms in females but not in males (Beydoun et al., 2013; Colangelo et al., 2009; Lucas et al., 2011; Persons et al., 2014; Tsujiguchi et al., 2019), and the higher prevalence of depression in females is related to their susceptibility to n-3 PUFA insufficiency (Colangelo et al., 2009). These inconsistencies could be partly attributed to the difference in sample size between the males (*n* = 48) and females (*n* = 91) in our study. In addition, most of the observational studies have collected data from the general population instead of depressed patients. Moreover, in previous epidemiological research, fatty acid composition was evaluated by using the validated food frequency questionnaire, which might be less representative. Currently, the associations between n-3 PUFAs and depressive symptoms remain controversial. Several negative results have also been reported in observational or intervention studies (Murakami et al., 2008; Giles et al., 2013; Yang et al., 2022). Notably, dietary or nutritional supplemental n-3 PUFAs were proven to have many beneficial effects on the adjunctive treatment for depression. However, the treatment effect of n-3 PUFAs may be induced by an increase in the ratio of n-3/n-6 PUFAs, which further indicates the detrimental role of n-6 PUFAs in depression.

We also found that only-child status and positive family history are the risk factors for depression while being coupled is the protective factor in first-diagnosed, drug-naïve patients with MDD. These results are consistent with previous studies in China, in which females, single, having diabetes, experiencing negative life events, and lacking social support are risk factors for depressive symptoms among older Chinese adults (Qiu et al., 2020).

Several limitations also need to be acknowledged. This is a cross-sectional exploratory investigation, which could not conclude a causal relationship. Although the study included first-diagnosed patients who had never taken antidepressants, the potential impact of prior medication use on fatty acid composition is not considered. All the participants were recruited from the hospital and the participants who took fish oil or regularly consumed fatty fish were excluded, as a result, the sample included in our study may not be representative of the broader population. The results have a limited degree of external validity. The absence of data regarding changes in dietary preferences and physical activity levels among individuals with depression (Firth et al., 2018; Schuch et al., 2018; Pearce et al., 2022), which could potentially impact fatty acid status (Hodson et al., 2008; Nikolaidis and Mougios, 2004). Potential cultural or geographical implications on fatty acid composition and mental health should be taken into account in future research.

## 5 Conclusion

Our findings indicate higher levels of n-6 PUFAs and SFAs in female patients with depression and the relationship between fatty acid composition and depressive symptoms was more pronounced in females than males. We propose that examining the fatty acid composition in individuals with depression should consider gender distribution as a significant possible confounding factor. In addition, when patients with depression undergo adjunctive treatment by targeting fatty acid metabolism, they should consider sex difference as an important factor. In summary, investigating the sex differences in fatty acid composition in patients with depression provides valuable insights for novel therapeutics, facilitates personalized treatment, and promotes the development of precision medicine for psychiatric disorders.

## Data Availability

The original contributions presented in the study are included in the article/Supplementary Materials, further inquiries can be directed to the corresponding author.
